# Increased risk of adverse events following the treatment of associated versus elementary acetabular fractures: a matched analysis of short-term complications

**DOI:** 10.1007/s00402-024-05726-3

**Published:** 2024-12-18

**Authors:** Sarah Cole, Sarah Whitaker, Conor O’Neill, James Satalich, Brady Ernst, Le Kang, Rami Hawila, Jibanananda Satpathy, Stephen Kates

**Affiliations:** 1 Virginia Commonwealth University School of Medicine, Richmond, VA USA; 2 Department of Orthopaedic Surgery, Virginia Commonwealth University Health System, Richmond, VA USA; 3 Department of Orthopaedic Surgery, Duke University Health System, Durham, NC USA; 4 Department of Biostatistics, Virginia Commonwealth University School of Public Health, Richmond, VA USA

**Keywords:** ORIF, Associated acetabular fracture, Elementary acetabular fracture, NSQIP

## Abstract

**Purpose:**

This retrospective cohort study aims to compare short-term complication rates between patients receiving open reduction and internal fixation (ORIF) for associated versus elementary acetabular fractures, with a secondary objective of identifying independent risk factors for adverse outcomes.

**Methods:**

The American College of Surgeons (ACS) National Surgical Quality Improvement Program (NSQIP) database was queried using current procedural terminology (CPT) codes to identify patients that underwent ORIF for associated acetabular (CPT 27228) or elementary acetabular fractures (CPT 27226, 27227) from 2010 to 2021. Propensity score matching was employed to account for baseline differences and the short-term complication rates were compared between the cohorts.

**Results:**

We identified 1,330 patients who underwent ORIF for an acetabular fracture between 2010 and 2021, including 868 patients with elementary fractures and 462 with associated fractures. After matching, there were 462 patients in each cohort. The matched associated acetabular fracture group experienced higher rates of any adverse event (AAE, p = 0.029), transfusion (p = 0.010), and reoperation (p = 0.049). Increased age, increased operative time, higher body mass index (BMI), and prolonged hospital length of stay (LOS) augmented the risk of any complication.

**Conclusion:**

The findings of this study identify areas of greater risk of postoperative complications after ORIF in individuals with associated versus elementary acetabular fractures. Discussion of these heightened risks is critical to appropriate patient care. Understanding these risks plays an important role in the clinical decision-making process and may identify areas to monitor patients postoperatively.

## Introduction

Acetabular fractures are rare injuries which pose complex challenges for surgeons, representing 2–8% of all fractures and occurring with an incidence of only 3 per 100,000 patients per year [1, 2]. Due to the complex presentation of these fractures and high associated mortality [3], surgical reduction is typically favored over nonoperative treatment due to lower mortality and in-hospital complication rates [4, 5]. Recent estimates show that 44–77% of patients with acetabular fractures are treated operatively [1, 6], with the preferred treatment being open reduction and internal fixation (ORIF) [7–9]. Despite improved outcomes with ORIF, patients are still at risk for postoperative complications, including infection, bleeding, heterotopic ossification, avascular necrosis, nerve palsy, conversion to total hip replacement, and death [10–14]. In addition, recent studies have shown that such complications occur more frequently in older patients [5, 15, 16]. Given the progressively aging population of patients with acetabular fractures [17–20], updated research is needed to assess the risk of postoperative complications for patients undergoing ORIF for acetabular fractures.

In 1961, Letournel proposed a classification system for acetabular fractures that identified five elementary fracture patterns (posterior wall, posterior column, anterior wall, anterior column, and transverse fractures) and five associated fracture patterns (T-type, posterior column and posterior wall, transverse and posterior wall, anterior column/wall and posterior hemitransverse, and both-column fractures) [21]. Using this classification system, Kelly, et al. found that associated fracture patterns occur more frequently than elementary fractures, representing 60.2% of all acetabular fractures [14]. This classification system has important clinical relevance, as fracture type may influence outcomes and adverse event occurrence. Several recent studies have found that fractures involving, but not necessarily limited to, the posterior wall are associated with poor outcomes and worse survivorship when compared to all other fracture patterns [5, 11, 22–24]. In addition, associated acetabular fractures may have lower rates of anatomic reduction following operative treatment when compared to elementary fractures [11–13, 25]. While current evidence suggests that associated acetabular fractures confer worse postoperative outcomes when compared to elementary fractures, there is limited contemporary literature comparing short-term complication rates between these two groups. Mears et al. and Matta et al. found worse survivorship and lower rates of successful reduction after ORIF in patients with associated acetabular fracture patterns compared to elementary fracture patterns, though Mears’s analysis is limited to 1996 and Matta’s is limited to 2003 [12, 13]. Kojima et al. found worse outcomes in patients with associated fracture patterns, but this study only included 137 patients from a single center [23]. Therefore, there is a need for updated research comparing short-term complications in patients receiving ORIF for associated and elementary acetabular fracture patterns in a larger sample size.

Our study addresses these limitations by providing a larger sample size across hundreds of institutions. Our primary objective is to compare short-term complication rates, reoperation, extended length of stay, and mortality after ORIF in patients with elementary and associated pattern acetabular fractures. We expect to find worse overall outcomes in patients with associated pattern fractures due to the complex nature of these injuries. A secondary objective is to determine if there are any independent risk factors which may predict the occurrence of any adverse event postoperatively.

## Materials and methods

The American College of Surgeons (ACS) National Surgery Quality Improvement Program (NSQIP) database was queried to identify relevant cases for this retrospective cohort study. The NSQIP database prospectively collects surgical data from participating institutions. Variables include preoperative characteristics, intraoperative variables, and rates of several complications, which are monitored for 30 days postoperatively. The most recent iteration (2021) consists of 983,851 cases from 685 institutions and includes 260 variables [26]. Participating institutions are subject to random audits which have found inter-rater disagreement rates of approximately 2% [27–29].

Patients were identified who underwent open reduction and internal fixation (ORIF) for treatment of elementary pattern acetabular fractures (CPT 27226 and 27227) or of associated pattern acetabular fractures (CPT 27228) between 2005 and 2021. Cases were included if the appropriate current procedure terminology (CPT) was designated as either the primary or an additional procedure in order to find all relevant patients. Cases were excluded if they appeared in both the elementary and associated pattern fracture groups. Cases were additionally excluded if they had unknown or null values for sex, body mass index (BMI), functional status, American Society of Anesthesiologists (ASA) classification, operative time, anesthesia technique, or total length of hospital stay. Once the relevant cases for both the elementary and associated fracture pattern cohorts were identified, preoperative characteristics and outcome data were collected for each participant. Cohorts underwent 1:1 propensity match for age, BMI, sex, race, smoking status, ASA classification, functional status, time from hospital admission to operation, preoperative steroid use, and history of diabetes, hypertension requiring medication, congestive heart failure (CHF), chronic obstructive pulmonary disorder (COPD), or any bleeding disorder. The inclusion of these factors in the matching model maximized the model’s power without compromising the number of patients in each cohort.

Outcome data was collected for each matched patient and included death within 30 days, surgical site infection, wound dehiscence, acute renal failure requiring dialysis, sepsis, pulmonary embolism, myocardial infarction, cardiac arrest, unplanned intubation, postoperative transfusion, deep vein thrombosis (DVT), pneumonia, urinary tract infection (UTIs), return to the OR, extended length of stay (LOS), and any adverse event (AAE). Extended LOS was defined as total length of hospital stay greater than the pre-match mean hospital stay across both cohorts. AAE included any occurrence of surgical site infection, wound dehiscence, sepsis, unplanned intubation, postoperative transfusion, pneumonia, DVT, pulmonary embolism, UTI, stroke, cardiac arrest, myocardial infarction (MI), return to the OR, and death within 30 days.

Statistical analysis was performed using RStudio software version 2023.06.1 + 524 (R Foundation for Statistical Computing, Vienna, Austria). Propensity score matching using the nearest neighbor method was employed to match cohorts. To compare baseline demographics and characteristics between the two groups, i.e., elementary versus associated pattern acetabular fractures group, the Wilcoxon rank sum test and the chi-squared test were used for continuous and categorical variables, respectively. The multivariable logistic regression model was employed to identify risk factors for any adverse event (AAE) using those variables which were found to be statistically significant from two group comparisons. The multivariate odds-ratio logistic regression analysis was also employed not only to model the associations between risk factors and marginal probabilities for binary outcomes, but also to model the associations between risk factors and pairwise dependency among binary outcomes in terms of odds ratios (OR) [30]. In order to avoid model redundancy, an ad hoc screening for correlated binary outcomes with high percent agreement values was performed. Statistical significance was defined as p < 0.05 throughout our analyses.

## Results

### Demographics

In total, 1,330 patients were identified to have undergone an ORIF for an acetabular fracture with 868 (65.3%) being treated for an elementary acetabular fracture and 462 (34.7%) for an associated acetabular fracture. After propensity score matching, a total of 924 patients were included with 462 patients in both the elementary fracture and associated fracture groups.

In the unmatched group analysis, there were no statistically significant differences in sex, length of stay, outpatient status, ASA classification, dependent functional status, smoking status, and comorbidities including CHF, dialysis, steroid use, bleeding disorders, ascites, preoperative transfusion, diabetes, and COPD. There were statistically significant differences in age, BMI, operative time, length of stay (LOS), and time from admission to operationwith all of the listed characteristics being significantly greater in the associated fracture group except for BMI (Table 1).

**Table 1:** Demographic and comorbidity characteristics for patients undergoing open reduction and internal fixation (ORIF) for treatment of elementary and associated acetabular fractures

	Elementary Unmatched (%)	Associated Unmatched (%)	p-value	Elementary Matched (%)	Associated Matched (%)	p-value
Patients, N (%)	868 (65.3)	462 (34.7)		462 (50)	462 (50)	
Age (years, mean ± SD)	62.2 ± 20.5	65.8 ± 18.9	**0.002**	66.6 ± 18.8	65.8 ± 18.9	0.594
BMI (kg/m^2^, mean ± SD)	27.5 ± 7.35	26.6 ± 6.96	**0.037**	26.8 ± 6.87	26.6 ± 6.96	0.910
Male sex (%)	455 (52.4)	235 (50.9)	0.630	229 (49.6)	235 (50.9)	0.742
Operative time (mins)	156 ± 90.0	192 ± 111	**< 0.001**	153 ± 91.6	192 ± 111	**< 0.001**
Length of stay	7.29 ± 6.85	7.80 ± 5.63	**< 0.001**	7.74 ± 6.65	7.80 ± 5.63	0.300
Outpatient status	26 (3.0)	8 (1.7)	0.227	7 (1.5)	8 (1.7)	1.000
ASA class			0.484			0.919
1 (no disturbance)	59 (6.8)	25 (5.4)	–	25 (5.4)	25 (5.4)	–
2 (mild disturbance)	298 (34.3)	146 (31.6)	–	141 (30.5)	146 (31.6)	–
3 (severe disturbance)	409 (47.1)	234 (50.6)	–	232 (50.2)	234 (50.6)	–
4 (life-threatening disturbance)	102 (11.8)	57 (12.3)	–	64 (13.9)	57 (12.3)	–
5 (moribund)	0	0	–	0	0	–
Race			0.311			0.767
White	638 (73.5)	346 (74.9)	–	351 (76.0)	346 (74.9)	–
Black	73 (8.4)	40 (8.7)	–	37 (8.0)	40 (8.7)	–
Asian	15 (1.7)	9 (1.9)	–	9 (1.9)	9 (1.9)	–
Other	142 (16.4)	67 (14.5)	–	65 (14.1)	67 (14.5)	–
Dependent functional status (partial or total)	82 (9.45)	47 (10.2)	0.742	47 (10.2)	47 (10.2)	1.000
Current smoker	161 (18.5)	96 (20.8)	0.364	89 (19.3)	96 (20.8)	0.622
Comorbidities N (%)						
Congestive heart failure	21 (2.4)	11 (2.4)	1.000	12 (2.6)	11 (2.4)	1.000
Dialysis	10 (1.2)	3 (0.6)	0.552	5 (1.1)	3 (0.6)	0.723
Steroid use	42 (4.8)	16 (3.5)	0.304	27 (5.8)	16 (3.5)	0.118
Bleeding disorder	81 (9.3)	51 (11.0)	0.371	50 (10.8)	51 (11.0)	1.000
Ascites	3 (0.3)	0 (0)	0.510	1 (0.2)	0 (0)	1.000
Preoperative transfusion	42 (4.8)	31 (6.7)	0.194	23 (5.0)	31 (6.7)	0.326
Diabetes	127 (14.6)	70 (15.2)	0.862	71 (15.4)	70 (15.2)	0.746
IDDM	65 (7.5)	29 (6.3)	–	25 (5.4)	29 (6.3)	–
NIDDM	62 (7.1)	41 (8.9)	–	46 (10.0)	41 (8.9)	–
COPD	56 (6.5)	38 (8.2)	0.276	38 (8.2)	38 (8.2)	1.000
Days from hospital admission to operation	1.86 ± 2.24	2.19 ± 2.10	**< 0.001**	2.20 ± 2.51	2.19 ± 2.10	0.247

All bolded values were deemed statistically significant with statistical significance defined as P < 0.05

*BMI* body mass index, *COPD* chronic obstructive pulmonary disease, *Dialysis* acute or chronic renal failure requiring dialysis within 2 weeks of indexed procedure, *IDDM* insulin-dependent diabetes mellitus, *NIDDM* non-insulin-dependent diabetes mellitus

After propensity score matching, only operative time remained significantly different between cohorts. In the elementary fracture cohort, the average age was 66.6 ± 18.8 years, the mean BMI was 26.8 ± 6.87 kg/m^2^, and the percentage of males was 49.6% (n = 229). In the associated fracture cohort, the respective values were 65.8 ± 18.9 years, 26.6 ± 6.96 kg/m^2^, and 50.9% males (n = 235). Further demographic data are tallied in Table 1.

### Outcomes

Before matching, there was a significantly greater risk of AAE, transfusion, wound dehiscence, and pneumonia postoperatively in the associated fracture cohort (Table 2). After matching, the associated fracture cohort had a significantly higher rate of AAE (49.4% vs. 42%, p = 0.029), transfusion (42.6% vs. 34.2%, p = 0.010), and reoperation (6.49% vs. 3.46%, p = 0.049) following the ORIF procedure (Table 2). Other complications including death, sepsis, pulmonary embolism, acute renal failure, MI, cardiac arrest, stroke, DVT, UTI, intubation issues, surgical site infection (SSI), and extended LOS were not found to vary significantly between the two groups both prior to and following matching.

**Table 2:** Incidence of adverse events for patients undergoing ORIF for elementary vs. associated acetabular fractures

	Elementary Unmatched		Associated Unmatched		p-value	Elementary Matched		Associated Matched		p-value	Overall Matched	
	No	Rate (%)	No	Rate		No	Rate	No	Rate		No	Rate (%)
Any adverse event	325	37.4	228	49.4	**< 0.001**	194	42.0	228	49.4	**0.029**	422	45.7
Death	25	2.9	16	3.5	0.675	14	3.0	16	3.5	0.853	30	3.2
Wound dehiscence	1	0.1	5	1.1	**0.038**	0	0	5	1.1	0.073	5	0.5
Sepsis	11	1.3	11	2.4	0.197	5	1.1	11	2.4	0.207	16	1.7
Pulmonary embolism	11	1.3	13	2.8	0.072	7	1.5	13	2.8	0.258	20	2.2
Acute renal failure	1	0.1	3	0.6	0.243	0	0	3	0.6	0.247	3	0.3
Myocardial infarction	7	0.8	4	0.9	1.000	4	0.9	4	0.9	1.000	8	0.9
Cardiac arrest	4	0.5	2	0.4	1.000	1	0.2	2	0.4	1.000	3	0.3
Stroke	1	0.1	1	0.2	1.000	1	0.2	1	0.2	1.000	2	0.26
Transfusion	258	29.7	197	42.6	**< 0.001**	158	34.2	197	42.6	**0.010**	355	38.4
DVT	23	2.6	17	3.7	0.380	11	2.4	17	3.7	0.337	28	3.0
UTI	25	2.9	7	1.5	0.174	14	3.0	7	1.5	0.185	21	2.3
Pneumonia	11	1.3	15	3.2	**0.023**	8	1.7	15	3.2	0.205	23	2.5
Intubation issues	10	1.2	8	1.7	0.534	3	0.6	8	1.7	0.225	11	1.2
SSI	25	2.9	18	3.9	0.404	12	2.6	18	3.9	0.353	30	3.2
Return to the OR	35	4.0	30	6.5	0.065	16	3.5	30	6.5	**0.049**	46	5.0
Extended LOS	84	9.7	50	10.8	0.572	46	10.0	50	10.8	0.746	96	10.4

Any adverse event (AAE): superficial and deep surgical site infection, organ space infection, wound dehiscence, acute renal failure, intubation (fail to wean or reintubation), post-operative transfusion, pneumonia, DVT, PE, UTI, stroke, cardiac arrest, MI, return to the OR, death

All bolded values were deemed statistically significant with statistical significance defined as P < 0.05

*DVT* deep vein thrombosis, *UTI* urinary tract infection, *SSI* surgical site infection, *LOS* length of stay (extended: greater than 1 standard deviation above the mean), *OR* operating room, *Intubation issues* re-intubation or failure to wean from intubation

When accounting for significant patient demographics and procedure characteristics variables identified in Table 1, increased age (in one-year intervals) (OR: 1.045, 95% CI 1.037–1.053), increased operative time (in one minute intervals) (OR: 1.008, 95% CI 1.006–1.009), and increased length of hospital stay (in one day intervals) (OR: 1.111, 95% CI 1.076–1.147) were found to significantly increase the risk for AAE (Table 3). The associated fracture has a higher odds of developing AAE as compared to elementary fracture but its OR was not significantly different from 1 (Table 3).

**Table 3:** Odds ratios of developing any adverse event (AAE) post-surgery as related to significant patient demographics and procedure characteristics based on unmatched data

	Odds ratio	95% CI	p-value
Age (1 year intervals)	1.045	1.037–1.053	**< 0.001**
BMI	1.012	0.994–1.030	0.188
Fracture type			
Elementary	Reference	–	–
Associated	1.191	0.914–1.550	0.195
Operative time (1 min intervals)	1.008	1.006–1.009	**< 0.001**
LOS (1 day intervals)	1.111	1.076–1.147	**< 0.001**
Time from hospital admission to operation (1 day intervals)	0.949	0.883–1.020	0.158

Significant variables from Table 1 were included in a multivariable logistic regression model for predicting AAE

All bolded values were deemed statistically significant with statistical significance defined as P < 0.05

*LOS* length of stay, *CI* confidence interval

A bivariate logistic regression modeling result for transfuse and extended length of stay (LOS) accounting for significant patient demographics and procedure characteristics was reported in Table 4. The reason for choosing these two AE outcomes is twofold: the percentage agreement below 66% between these two binary outcomes, one of the lowest between any binary outcome pair, indicated moderate information overlapping; and multivariate modeling is feasible and potentially more powerful if there are relatively large numbers of events (Table 2). Increased age was associated with higher risk for transfusion (OR: 1.048, 95% CI 1.039–1.056), however, the odds ratio for transfusion for subjects with extended LOS versus subjects without extended LOS was 5% lower (OR: 0.950, 95% CI 0.913–0.989) given subjects 1 year older, indicating that as age increases, the risk of subjects with extended LOS getting transfusion was slightly lower as compared to the risk of subjects without extended LOS getting transfusion. Increased operative time (OR: 1.009, 95% CI 1.007–1.010) was associated with increased risk for transfusion. One more day of hospital stay was associated with increased risk for transfusion (OR: 1.052, 95% CI 1.018–1.087) and increased risk for extended LOS (OR: 1.710, 95% CI 1.573–1.859).

**Table 4:** Odds Ratios of transfusion and extended length of stay as related to significant patient demographics and procedure characteristics based on unmatched data

	Odds ratio	95% CI	p-value
Age: 1	1.048	1.039–1.056	**< 0.001**
Age: 2	0.990	0.975–1.006	0.237
Age: 3	0.950	0.913–0.989	**0.013**
BMI: 1	1.002	0.983–1.021	0.848
BMI: 2	0.998	0.962–1.035	0.904
BMI: 3	0.956	0.879–1.041	0.304
Associated vs elementary: 1	1.262	0.964–1.653	0.091
Associated vs Elementary: 2	1.011	0.563–1.818	0.970
Associated vs elementary: 3	0.844	0.228–3.125	0.800
Operative time: 1	1.009	1.007–1.010	**< 0.001**
Operative time: 2	0.997	0.994–1.000	0.084
Operative time: 3	0.997	0.989–1.005	0.424
LOS (1 day intervals): 1	1.052	1.018–1.087	**0.002**
LOS (1 day intervals): 2	1.710	1.573–1.859	**< 0.001**
LOS (1 day intervals): 3	0.838	0.687–1.023	0.082
Admission to operation: 1	1.006	0.935–1.081	0.880
Admission to operation: 2	0.984	0.881–1.100	0.781
Admission to operation: 3	1.181	0.904–1.544	0.223

Rows with variable names followed by 1 are for marginal odds ratios for transfusion, rows with variable names followed by 2 are for marginal odds ratios for extended length of stay (LOS), and rows with variable names followed by 3 are for dependency associations in terms of odds ratios. [50]

All bolded values were deemed statistically significant with statistical significance defined as P < 0.05

## Discussion

This study found a significantly greater risk of developing any adverse event following an ORIF of an associated acetabular fracture when compared to ORIF of an elementary acetabular fracture. In particular, associated fracture pattern patients demonstrated a higher rate of postoperative transfusion and need for reoperation even after accounting for age, sex, BMI, race, ASA classification, and other demographic variables. These findings are consistent with our initial hypothesis that associated fracture patterns will increase the risk of postoperative short-term complications and are aligned with current evidence surrounding treatment of acetabular fractures.

The finding of increased risk of adverse events in individuals with associated fracture patterns has been corroborated by prior research. Multiple studies analyzing postoperative outcomes of ORIF for acetabular fractures have also found greater risks of poor outcomes with associated fracture patterns when compared to elementary acetabular fractures [5, 12, 13, 16, 22–24, 31–33]. While some reported on overall worse satisfaction and functional outcomes in patients with associated fractures [16, 23, 31, 33], many others have cited associated fractures as a risk factor for malreduction with one study finding that 100% of patients with malreductions post-ORIF had initially sustained an associated acetabular fracture [5, 12, 13, 24, 32]. Malreduction has been identified in multiple studies as a major risk factor for poor surgical outcomes including reoperation, functional abilities, and infection [4, 13, 32, 34]. Furthermore, Cichos et al. found higher rates of conversion to THA in patients undergoing an ORIF for associated acetabular fractures, and Kreder et al. identified higher instances of osteoarthritic changes in patients with prior associated acetabular fractures [22, 31]. Moreover, other studies have cited an increased rate of concomitant injuries in associated acetabular fracture cases than in elementary acetabular fracture cases [23, 35]. These concurrent injuries were most typically found in the lower limbs and head[14, 24] and were commonly associated with increased risk of postoperative complications and malreduction [24, 32]. Therefore, polytrauma cases and worse reduction outcomes may in part explain the significant increase in short-term complications following the reduction of associated acetabular fractures. By incurring greater risk through additional traumas, our findings of increased short-term complications in associated fracture patients align with prior studies on ORIF outcomes. However, not all prior studies are in agreement with our findings. Moed et al. reported that functional outcome scores were not significantly affected by fracture type or the presence of concomitant injuries [33]. Kreder et al. additionally found no difference in conversion to THA based on initial acetabular fracture type, and Matta et al. and Sanders et al. concluded that the fracture pattern is not a good prognostic indicator of long-term hip outcomes [12, 31, 34]. Additionally, Liebergall et al. and Matta et al. did not find any significant association between concomitant injuries and worse outcomes [12, 16]. Though these studies contradict prior research outlined in the previous paragraph, they did not specifically assess short-term complication rate differences among fracture types and are focused more on long term patient reported outcome measures. Moreover, of the studies that found contradictory conclusions to our own, only Kreder et al. completed a matched cohort study but only included age and sex in their propensity score matching. The use of propensity score matching with the inclusion of a wide range of demographic variables adds validity to our results and helps prevent confounding variables from influencing statistical significance.

Looking at specific adverse events, transfusion wasfound to occur at higher rates in patients undergoing ORIF for associated acetabular fractures. The significance of the injury as well as the surgical procedure have been shown to lead to substantial blood loss in some cases that may be as high as 1232–2818 mL [36–38]. Additionally, the most common injuries accompanying acetabular fractures are lower limb injuries [14, 24], and fractures of the femoral shaft are correlated with high blood loss [38–40]. Given that associated fracture patterns tend to occur at a higher rate with concomitant injuries [23, 35], there could be a relationship between the significance of concomitant injuries in polytrauma cases involving associated acetabular fractures and the greater risk of postoperative transfusion. On top of an injury already related to high blood loss and a riskier surgical procedure, there may be other injuries present that exacerbate blood loss in these patients. Unfortunately, recognizing this difference in blood loss based on fracture pattern will not alter clinical management of these patients outside of possibly increasing vigilance for evidence of severe hemorrhaging. In general, researchers have been looking to identify ways to minimize blood loss in these trauma patients and have seen mixed evidence for the efficacy of tranexamic acid in acetabular patients [41, 42]. Despite its benefits in total joints patients, tranexamic acid was not found to significantly reduce the need for transfusion rates in patients with acetabular fractures in Wadhwa et al.[42] At present, the best strategy to minimize blood loss and reduce mortality in both elementary and associated acetabular fracture patients is to use a multidisciplinary approach to conserve blood and to intervene as early as possible if concern for hemorrhaging arises [43]. Overall, short-term complication rates between elementary and associated acetabular fractures are rarely examined in recent studies and offer many opportunities for future research.

Along these lines, reoperation is another complication with significantly greater rates following surgical care of an associated acetabular fracture. Several studies have shown malreduction to be a significant risk factor for poor long-term hip survivorship, conversion to THA, worse functional outcomes, and reoperation [4, 5, 13, 32]. Though malreduction itself was not a variable involved in this study, several recent studies have shown increased incidence of malreduction in patients with associated fractures compared to elementary fracture patterns. Our finding of increased return to OR rates in patients with associated fractures is, therefore, in line with studies that show higher rates of reoperation in fractures with malreduction. Ding et al. identified delayed time from hospital admission to operation as a significant risk factor in reoperation rates after ORIF for acetabular fractures due to risk of infection [44]. While time from admission to surgery was significantly increased in the unmatched associated fracture cohort, the matched cohorts eliminated this variation between fracture types by including time from admission to surgery as a matched variable. Thus, the significant difference between reoperation rates between fracture types cannot be concluded as solely due to delays in surgical intervention between the groups. Additionally, no significant differences in postoperative infection rates were identified between associated and elementary fracture preoperative patterns so our finding of increased rate of reoperation cannot be explained by increased infection rates. Ding et al. further concluded that fracture patterns and concomitant injuries were not significantly correlated with early revision reoperations [44]. However, they did not specifically compare associated to elementary fracture patterns and instead looked at each fracture type individually. Grouping the fractures into these two major cohorts may have revealed patterns in complication rates not found by looking at each fracture type in isolation.

In addition to associated fracture patterns as a risk factor for poor surgical outcomes, our study also identified prolonged hospital stay, longer time to surgery, increased age, greater BMI, and higher operative time as risk factors for any postoperative adverse event. In the majority of prior studies, researchers have concluded that age has a significant impact on surgical outcomes [5, 12, 16, 23, 31, 32]. Of these studies, several demonstrated a significant relationship between older patients and an increased risk of malreduction[12, 23, 32] while others found that age correlated with worse functional outcomes and long-term hip survivorship [5, 16, 31]. Moreover, a prolonged period between hospital admission and surgery was found to additionally be connected to worse reduction outcomes, functional scores, and infection rates [12, 23, 25, 44]. In this context, our finding that age and prolonged time to surgery were correlated with significantly higher complication rates may also relate to the reduction outcome of the procedure. As we did not assess long-term complications and outcomes, the other prior findings are not able to be corroborated or negated by this study. Similarly, several studies have also reached the conclusion that sex does not have a significant role in short and long-term surgical outcomes [32–34]. Not all prior research agrees with our conclusions on preoperative risk factors that correlate with postoperative complications. Both Kojima et al. and Lundin et al. found male sex to be a protective factor postoperatively, particularly when assessing reoperation rate [23, 45]. However, Lundin et al. also included individuals undergoing THA as the primary treatment for acetabular factors which may have impacted their results [45]. Additionally, Sanders et al. found that ASA classification only predicted surgical outcomes until they completed a multivariate analysis and did not find age to be a significant predictor of prognosis [34]. However, Sanders et al. only focused on elderly patients and did not include any individuals below the age of 60 years old. Prior studies that have attributed better postoperative outcomes to younger patients often did so in individuals below their included sample age restrictions.

While the study yielded significant results that align with previous literature on acetabular fractures, there were several limitations in our research. Though the NSQIP database provides a wealth of information on postoperative short-term outcomes and patient demographics, the database is designed to account for results of any surgical specialty and does not include variables specific to orthopedic outcomes including quality of fracture reduction, radiographic results, and musculoskeletal functional assessments. Several of these outcomes were assessed in prior research on ORIF procedures with multiple studies finding a higher risk of worse functional and unsatisfactory reduction results following an ORIF for associated acetabular fractures [12, 13, 24, 25, 32, 33]. However, it may still be possible to infer some of this information using the NSQIP reports of reoperation rate as malreduction is a common cause for returning to the OR shortly after an ORIF [45, 46]. Similarly, the NSQIP database also did not provide rates of nerve injury following the procedure which is one of the most common adverse events after surgical management of acetabular fractures with one studying finding the rate to be 12% after any surgical treatment [45]. This limits our ability to further analyze differences in complication rates based on acetabular fracture pattern prior to surgical intervention. Lastly, the database used only accounts for complications occurring within 30 days of the operation and does not provide information on long-term implications of these procedures on patient health and wellbeing. This limitation prevents our studying from examining the long-term survival of the injured hip as acetabular fractures have been shown to correlate with post-traumatic osteoarthritic changes in up to 57% of patients [12, 21, 34, 47–50]. Furthermore, Ding et al. found that the majority of revision reoperations occur at an average of 41 weeks, a time period not included in this study [44]. Lastly, some of the patients included in this study may have attended follow-up visits at a non-NSQIP institution resulting in the loss of their complication data. Though the data collected has limitations, the validity of the data used in combination with our inclusion criteria, use of propensity match scores, and large sample size add considerable validity to research into a question with few recent comparative studies. Current literature that directly compares outcomes between associated and elementary acetabular ORIFs is limited, particularly in recent years, and this study helps to fill the gap in research.

## Conclusion

In conclusion, this study identified a significantly greater risk of any adverse event following an ORIF for patients that have sustained an associated acetabular fracture compared to patients with an elementary acetabular fracture, especially in terms of transfusion and reoperation. Prior studies have come to similar conclusions and have indicated that these differences may at least in part be related to higher rates of malreduction and additional injuries in this patient cohort. While ORIF remains a validated and crucial treatment plan for patients with complex acetabular fractures, this study contributes to the discussion on overall risk for these patients and identifies areas to be mindful of in the surgical planning process for treatment of both associated and elementary acetabular fractures.

## Data Availability

De-identified data is available upon request from researchers at institutions affiliated with the National Surgical Quality Improvement Program (NSQIP) in compliance with and subject to the data use agreement with the American College of Surgeons (ACS).
